# The CC Genotype of *Insulin-Induced Gene 2 rs7566605* Is a Protective Factor of Hypercholesteremia Susceptible to Mild Cognitive Impairment, Especially to the Executive Function of Patients with Type 2 Diabetes Mellitus

**DOI:** 10.1155/2020/4935831

**Published:** 2020-06-10

**Authors:** Haoqiang Zhang, Rong Huang, Sai Tian, Ke An, Wenwen Zhu, Jijing Shi, Wuyou Cao, Shaohua Wang

**Affiliations:** ^1^Department of Endocrinology, Affiliated Zhongda Hospital of Southeast University, No. 87 Dingjiaqiao Road, Nanjing, China 210009; ^2^School of Medicine, Southeast University, Nanjing, China 210009

## Abstract

**Methods:**

233 T2DM patients with MCI or without MCI were recruited. Baseline data and genotype frequency were compared between MCI and non-MCI groups. Demographic parameters and neuropsychological tests results were analyzed among patients with different genotypes. Further correlation and regression analysis were conducted to find the association between cognition and cholesterol.

**Results:**

Despite no significant statistical difference was detected, we observed higher levels of total cholesterol (TC) and low-density lipoprotein cholesterol (LDL) in patients with MCI than those without MCI. In addition, we observed higher TC and LDL levels in patients with GG or GC genotypes than those with CC genotype (*P* < 0.001, *P* = 0.004, or *P* < 0.001, *P* = 0.002). Interestingly, increased MoCA and decreased TMTB scores were found in patients with CC genotype, compared to those with GG or CG genotype (*P* = 0.009, *P* = 0.024, or *P* = 0.005, *P* = 0.109). Moreover, partial correlation (*P* = 0.030 and *P* = 0.004, respectively) and multiple linear regression (*P* = 0.030 and *P* = 0.005, respectively) showed that TC and LDL levels are associated with the TMTB score, indicating the executive function.

**Conclusions:**

CC genotype of *INSIG-2 rs7566605* may be a protective factor of hypercholesteremia susceptible to MCI, especially to the executive function of T2DM. This trial is registered with ChiCTROCC15006060.

## 1. Introduction

The prevalence of type 2 diabetes mellitus among adults has increased to 10.4% and affected 300 million worldwide [1]. Diabetes increased about 50% risk of Alzheimer's disease (AD) [2] with a prodromal stage called MCI, a transitional stage between normal cognition and AD dementia [3]. Previous researches suggested that uncontrolled hyperglycemia is a risk factor of T2DM with MCI [4, 5]. Epidemiological evidence showed that cholesterol is also a risk factor for AD [6]. In addition, cholesterol homeostasis failure is a cause of synaptic degeneration [7]. Our recent study demonstrated that poorly controlled cholesterol is associated with cognitive impairment in T2DM human [8]. Moreover, increased LDL cholesterol caused cognitive decline in *LDL^−/−^* mice [9]. So, we guess that the reason causing hypercholesterolemia may result in cognitive impairment.

*Insulin induced genes* (*INSIGs*), including *INSIG-1* [10] and *INSIG-2* [11], and sensors and mediators that regulate cholesterol homeostasis through sterol regulatory element-binding proteins (SREBPs) and SREBP cleavage-activating protein (SCAP), are discovered and regarded as crucial roles in cholesterol metabolism. *INSIGs* could negatively regulate 3-hydroxy-3-methylglutaryl coenzyme A reductase (HMG-CoA reductase), an important key enzyme of cholesterol synthesis [12]. So, we hypothesize that *INSIG-2* plays an important role in cognitive impairment via regulating cholesterol homeostasis.

There is an interesting SNP; *rs7566605*, which is located 10 kb upstream of *INSIG-2*, was found to be associated with obesity, assessed by BMI [13]. However, Wang et al. [14] had reported that *rs7566605* may be not associated with severe obesity in Chinese children. Although there was no association found between genotype at *rs7566605* and obesity-related phenotypes in this British Caucasian population [15], the promoter of *INSIG-2 rs7566605* SNP is associated with the prevalence of hypercholesterolemia [16]. Additionally, the CC genotype is an independent protective genetic factor for the progressing of hypercholesterolemia induced by high-fat diet, especially in female subjects. Zavattari et al. [17] conducted a study, which suggested that the *INSIG-2 rs7566605* SNP may play a role in metabolic complications related to obesity in obese children and adolescents. An intervention study showed that *rs7566605* SNP of *INSIG-2* had effects on weight change [18]. However, the relationship among *rs7566605* SNP of *INSIG-2*, hypercholesterolemia, and mild cognitive impairment remains unclear in patients with type 2 diabetes mellitus. In the present study, we aim to investigate the potential protective role of CC genotype of *INSIG-2* in cognitive decline.

## 2. Materials and Methods

### 2.1. Ethics

This present study was conducted in the Endocrinology Department, Affiliated Zhongda Hospital of Southeast University. All individuals were hospitalized patients and provided a written informed consent prior to the participation of this study. The present study was approved by the Research Ethics Committee, Affiliated Zhongda Hospital of Southeast University.

### 2.2. Subjects and Groups

This cross-sectional study recruited 233 (87 T2DM with MCI and 146 T2DM without MCI) hospitalized patients who satisfied the criteria of type 2 diabetes mellitus [19]. All individuals were right-handed Han Chinese having diabetes for more than 3 years. The diagnostic criteria for MCI were proposed by the MCI Working Group of the European Consortium for Alzheimer's Disease [20]. The individuals were included in or excluded from this study according to the criteria of our previous studies [21].

### 2.3. Clinical Data

The following demographic characteristics were gathered: age, gender, and education. Duration of diabetes mellitus (DM) and high blood pressure (HBP) were recorded. Medication histories of metformin and insulin were collected. Body mass index (BMI) was calculated. Fasting blood-glucose (FBG), glycosylated hemoglobin (HbA1c), triglyceride (TG), TC, LDL-C, and high-density lipoprotein (HDL-C), were determined from blood samples. The Laboratory Center of Zhongda Hospital implements internal and external quality control procedures as directed by the Chinese Laboratory Quality Control.

### 2.4. Neuropsychological Tests

Neuropsychological tests, including the Montreal Cognitive Assessment (MoCA), Digit Span Test (DST), Verbal Fluency Test (VFT), Clock Drawing Test(CDT), Trail Making Test-A (TMTA), Trail Making Test-B (TMTB), Auditory Verbal Learning test-immediate recall (AVLT-IR), Auditory Verbal Learning test-delayed recall (AVLT-DR), and logical memory test (LMT) were administered to evaluate patients' cognitive functions, such as semantic memory, episodic memory, attention, executive function, and visuospatial skills. MoCA was used to assess all cognitive ability, and these patients with T2DM were divided into MCI (<26) and non-MCI groups (≥26).

### 2.5. DNA Isolation

Genomic DNA was isolated from blood samples (EDTA treated) according to the protocol of a DNA purification kit (Puregene, Gentra System, Minneapolis, MN) and similar with our previous studies [22]. It can be described briefly as follows: 1 ml blood sample and 1 ml cell lysate solution (CL) were mixed sufficiently. After thorough mixing, the mixture is centrifuged for 2 minutes at 3600 rpm. Then, the supernatant was removed before 1.5 ml CL was added in the precipitate. The mixture was mixed and centrifuged for 2 minutes at 3600 rpm. Before 0.5 ml mixture (proteinase K: buffer PG = 1 : 120) was added in the precipitate, the supernatant was removed. The above mixture was vortex mixed immediately until the solution is free of lumps. And then, the mixture was incubated at 65°C for 30 minutes. After the mixture become green from red, 1 ml isopropanol was added in the mixture and mixed sufficiently until enough filamentous DNA appeared in the centrifuge tubes. Tubes with DNA were centrifuged for 10 minutes at 3600 rpm and then put them upside-down on clean absorbent paper for several minutes. After 1 ml 75% alcohol was added, the tubes were vortex mixed for 5 seconds and centrifuged for 3 minutes at 3600 rpm (this step was repeated 2 times). The tubes were put upside-down on a clean absorbent paper to dry for at least 5 minutes. One milliliter buffer TB was added to dissolve the DNA for 1 h at 65°C in the water bath. This dissolved DNA was used for further study.

### 2.6. *INSIG-2 rs7566605* SNP Measurements

The following forward primer sequence and reverse primer sequence were used. Forward primer sequence: 5′-ACGTTGGATGTCATTGCAATAGCCACTGCC-3′, reverse primer sequence: 5′-ACGTTGGATGAAAACCACCCTGGTACAGAC-3′. The DNA sequences of *INSIG-2* were amplificated in a reaction system (containing 172.25 *μ*l of 25 mM MgCl2, 53 *μ*l of 25 mM DNT Mix, 106 *μ*l of 5 U/*μ*l HotStar Taq, 331.25 *μ*l of ×10 PCR buffer, 10 ng/*μ*l of 1 well genomic DNA, 530 *μ*l of 0.5 *μ*mol/l primer, and 927.5 *μ*l HPLC grade water) and initiated at 94°C for 2 minutes, followed by 45 cycles of denaturation at 94°C for 20 seconds, 56°C for 30 seconds, and 72°C for 60 seconds and a final extension step at 72°C for 3 minutes. The above reaction products (9 *μ*l in total) were diluted 3 times and desalinated with resin. Desalted samples were placed on the sample target to crystallize naturally. Finally, mass spectrometry detection was performed.

### 2.7. Statistical Analysis

Statistical analyses were conducted with SPSS 20.0 (SPSS Inc., Chicago, IL, USA). Student's *t* test and one-way ANOVA were employed for normally distributed variables. The nonparametric Mann–Whitney *U* and Kruskal-Wallis tests were used for asymmetrically distributed variables. The chi-squared test was utilized to test for binary variables. Partial correlation analysis and multiple linear regression analysis were carried out to explore the relationships between the cognitive measures and demographic characteristics. All statistical significance was defined as *P* < 0.05.

## 3. Results

### 3.1. Demographic, Clinical, and Neuropsychological Data of T2DM Patients with or without MCI

In this cross-section study, demographic, clinical, and neuropsychological data were collected and are described in Table 1. There are significant differences in age, gender, education level, DM duration, HBP duration, and HbA1c levels in T2DM with or without MCI (all *P* < 0.05). However, we did not find significance between in BMI, smoking, insulin usage, metformin usage, FPG, TG, TC, HDL, LDL, ApoA1, ApoB, and LPa levels (all *P* > 0.05). As the important elements of cognition decline, MoCA, DST, VFT, CDT, AVLT-IR, AVLT-DR, and LMT scores decreased, while TMTA and TMTB scores increased in the MCI group, compared with the non-MCI group (all *P* < 0.05).

### 3.2. *INSIG-2 rs7566605* Genotype Frequencies between the MCI and Non-MCI Groups

No significant differences in *INSIG-2 rs7566605* genotype distributions were identified between the MCI and non-MCI groups (all *P* > 0.05, Table 2).

### 3.3. Demographic, Clinical, and Neuropsychological Data of T2DM Patients with Different Genotypes

Although there was no significant difference of genotype distribution in T2DM patients with or without MCI, we found difference of metformin use frequencies, TC, LDL, and ApoB levels, as well as MoCA score and TMTB score (Table 3, all *P* < 0.05), indicating the executive function. To further explore the role of *INSIG-2* genotypes in MCI, the values above were compared between each two groups with different genotypes. Interestingly, compared to the GG group, there are decreased TC and LDL levels in the GC group; however, we also detected lower TC, LDL, and ApoB levels in the CC group than that in the GG and GC groups (all *P* < 0.05). In addition, we measured a higher MoCA score in the CC group than the GC and GG groups (all *P* < 0.05). Furthermore, there is a decreased TMTB score in the CC group, compared to the GG group (*P* < 0.05) (Table 4).

### 3.4. Associations between TC, LDL and ApoB and Neuropsychological Test Scores

*INSIG-2* is an important gene associated with cholesterol metabolism. Improved cognition was found in CC genotype patients, compared with patients with GG and GC genotypes. To further investigate the potential mechanism between *INSIG-2* SNP and cognition decline, a partial correlation was administrated. TC and LDL are associated with TMTB score adjusted by age, education level, gender, DM duration, HBP duration, smoking, insulin usage, and metformin usage (*R* = 0.145, *P* = 0.030, and *R* = 0.193, *P* = 0.004, respectively) (Table 5).

### 3.5. Multiple Linear Regression Analysis with TMTB as the Dependent Variable

To further explore the potential role of TC and LDL in the cognition of T2DM patients, multiple linear regression analysis was performed. Corrected with age, education level, gender, DM duration, HBP duration, smoking, insulin usage, and metformin usage, TC and LDL are associated with TMTB, which is an index associated with executive function (*β* = 8.146, *P* = 0.030, and *β* = 13.704, *P* = 0.005, respectively) (Table 6).

## 4. Discussion

For the increased T2DM prevalence [1, 23], uncontrolled plasma glucose is a risk factor of AD and MCI in our previous studies [24–26] and others [27]. In the present study, our results supported these conclusions by discovering the increased HbA1c level of T2DM patients with MCI, compared to those without MCI. Although we found cognition decline in T2DM patients with hypercholesteremia in our previous study [8], and higher cholesterol levels in T2DM patients with MCI than those without MCI in the present research, the difference between levels of TC (4.72 ± 1.08 vs. 4.49 ± 1.02) or LDL (2.82, 2.34-3.68, vs. 2.71, 2.15-3.40) is not statistically significant. This may account for the limited participations. In addition, more and more research suggested that hypercholesteremia is a risk factor of cognitive impairment in several high-fat diet-induced or genetic hypercholesteremia animal studies [28–30] and in clinical trials [31, 32].

While the important role of cholesterol in the process of cognition decline is well recognized [33, 34], the role of cholesterol and the genetic factors regulating the metabolism of cholesterol in cognitive impairment with T2DM remains undiscovered. *INSIG-2* is a sensor and mediator that regulates the transcription and translation of HMG-CoA reductase and LDL receptor to keep cholesterol, especially LDL homeostasis [11]. The potential role of *INSIG-2* gene involved in T2DM with MCI is still unclear. Moreover, the promoter of *INSIG-2 rs7566605* SNP is associated with the prevalence of hypercholesterolemia [16]. Additionally, the CC genotype is an independent protective genetic factor for the progressing hypercholesterolemia induced by high-fat diet, especially in female subjects [17]. So, in this work, these genotypes of the participants were measured. We found more CC and less GG genotypes in T2DM patients without MCI than those with MCI, but there is no statistical significance (*P* = 0.056).

To further explore the relationship among *INSIG-2 rs7566605* SNP, hypercholesterolemia, and cognitive impairment, the level of demographic and clinical data and neuropsychological tests results were compared. Interestingly, we detected significant differences in TC, LDL, and ApoB levels as well as MoCA and TMTB scores among the three genotypes. In addition, decreased TC, LDL, and ApoB levels were observed in patients with CC genotype than these with GC or GG genotype. Moreover, increased MoCA score and decreased TMTB score were tested in patients with CC genotype, compared with those with GC or GG genotype. The above conclusions suggested that unbalanced TC and LDL result from ApoB and *INSIG-2 rs7566605* SNP may be associated with cognitive dysfunction of T2DM.

To investigate the association among TC, LDL, ApoB, and cognitive dysfunction, correlation analysis between these above cholesterol metabolic indexes and MoCA (or TMTB) scores was performed. Although we did not find a correlation between MoCA (or TMTB) and ApoB, significant correlations were found between TC or LDL and TMTB scores (*R* = 0.145, *P* = 0.030, or *R* = 0.193, *P* = 0.004, respectively), indicating the executive function [35]. Moreover, multiple linear regression with TMTB score insisted that TC and LDL are associated with the TMTB score independent from age, gender, education, BMI, DM duration, HBP duration, smoking, insulin, and metformin usage.

Overall, the *INSIG-2 rs7566605* SNP may be associated with cognitive decline. In other words, CC genotype has a neuroprotective effect on T2DM patients, especially for executive function. In addition, cholesterol homeostasis, especially for LDL, may be involved in these mechanisms.

In this study, we demonstrated lower TC and LDL levels and TMTB score in patients with CC genotype than those with GC or GG genotype. However, there is no significant difference of TMTB scores between patients with CC genotype and GC genotype (*P* = 0.109), due to the limited number of participants listed in this study. The present research showed decreased TC, LDL levels, and TMTB in the CC genotype, compared to those with other genotypes. Although previous studies suggested that *INSIG-2 rs7566605* SNP is associated with hypercholesteremia, we did not get the direct association between *INSIG-2 rs7566605* SNP and MCI. So, we just guess that CC genotypes may have the neuroprotective effects on T2DM patients. Furthermore, we only observed genotypes in this research; the expression level of *INSIG-2* was not detected. In our following study, we would like to measure the global expression level of *INSIG-2* in human and local *INSIG-2* in animal models. Last, our study is a cross-sectional study, which can only explain the association between *INSIG-2* genotype and mild cognitive impairment but fails to explain the causal relationship between them. The causal relationship between them needs to be further validated by a cohort study.

## Figures and Tables

**Table 1:** Demographic, clinical, and cognitive characteristics of T2DM patients with or without MCI.

	MCI (87)	Non-MCI (146)	*P*
Age (year)	60 (55-68)	59 (53-65)	0.046^‡^^∗^
Female (%)	44 (50.6)	47 (32.2)	0.005^§^^∗^
Education (year)	9 (9-12)	11 (9-12)	0.004^‡^^∗^
BMI (kg/m^2^)	24.486 (22.758-27.041)	24.671 (22.740-26.298)	0.730^‡^
DM duration (year)	12 (8-16)	10 (6-15)	0.025^‡^^∗^
HBP duration (year)	8 (0-17)	1 (0-10)	0.005^‡^^∗^
Smoking (%)	26 (29.9)	56 (38.4)	0.211^§^
Insulin usage (%)	50 (57.5)	86 (58.9)	0.783^§^
Metformin usage (%)	51 (58.6)	100 (68.5)	0.127^§^
HbA1c (%)	9.2 (8.0-10.8)	8.4 (7.4-10.0)	0.011^‡^^∗^
FPG (mmol/l)	7.80 (6.41-10.20)	7.60 (6.59-9.72)	0.382^‡^
TG (mmol/l)	1.85 (0.94-2.59)	1.45 (1.00-2.14)	0.100^‡^
TC (mmol/l)	4.72 ± 1.08	4.49 ± 1.02	0.149^†^
HDL (mmol/l)	1.09 (0.95-1.27)	1.10 (0.93-1.30)	0.686^‡^
LDL (mmol/l)	2.82 (2.34-3.68)	2.71 (2.15-3.40)	0.163^‡^
ApoA1	1.13 ± 0.29	1.11 ± 0.29	0.480^†^
ApoB	0.80 (0.66-0.90)	0.80 (0.64-0.96)	0.518^‡^
LPa	156 (83-315)	126 (70-270)	0.246^‡^
MoCA	23 (20-24)	27 (26-28)	<0.001^‡^^∗^
DST	11 (9-12)	12 (11-14)	<0.001^‡^^∗^
VFT	14 (13-17)	17 (14-21)	<0.001^‡^^∗^
CDT	3 (2-4)	4 (3-4)	<0.001^‡^^∗^
TMTA	70 (55-89)	55 (46-70)	<0.001^‡^^∗^
TMTB	187 (142-255)	131 (100-173)	<0.001^‡^^∗^
AVLT-IR	16 (13-18)	19 (16-23)	<0.001^‡^^∗^
AVLT-DR	5 (3-6)	6 (5-8)	<0.001^‡^^∗^
LMT	8 (4-10)	11 (8-14)	<0.001^‡^^∗^

BMI: body mass index; DM: diabetes mellitus; HBP: high blood pressure; FPG: fasting plasma glucose; TG: triglycerides; TC: total cholesterol; LDL: low-density lipoprotein cholesterol; HDL: high-density lipoprotein cholesterol; ApoA1: apolipoprotein A1; ApoB: apolipoprotein B; LPa: lipoprotein A; MoCA: Montreal Cognitive Assessment; DST: Digit Span Test; VFT: Verbal Fluency Test; CDT: Clock Drawing Test; TMTA: Trail Making Test-A; TMTB: Trail Making Test-B; AVLT-IR: Auditory Verbal Learning test-immediate recall; AVLT-DR: Auditory Verbal Learning test-delayed recall; LMT: logical memory test; MCI: individuals with mild cognitive impairment; Non-MCI: individuals without mild cognitive impairment. The data are presented as *n* (%), the mean ± SD, or the median (inter-quartile range) unless otherwise specified. ^†^Student's *t* test was employed for normally distributed variables. ^‡^The Mann–Whitney *U* test was employed for asymmetrically distributed variables. ^§^The chi-squared test was employed for categorical variables. ^∗^*P* < 0.05.

**Table 2:** Distributions of the *INSIG-2 rs7566605* genotypes in T2DM patients with MCI or normal cognition.

Genotypes	MCI (*n*, %)	Non-MCI (*n*, %)	OR (95% CL)	*P*
All	87	146		
G	114 (65.5)	165 (56.5)	1.000	
C	60 (34.5)	127 (43.5)	1.462 (0.991-2.158)	0.055
GG	38 (43.7)	51 (34.9)	1.000	
GC	38 (43.7)	63 (43.2)	1.235 (0.690-2.210)	0.476
CC	11 (12.6)	32 (21.9)	2.168 (0.971-4.841)	0.056
GC+CC	49 (56.3)	95 (65.1)	1.445 (0.839-2.487)	0.184

MCI: individuals with mild cognitive impairment; Non-MCI: individuals without mild cognitive impairment. The genotypes and allele frequencies were compared between the groups using Pearson's *χ*^2^ tests.

**Table 3:** Demographic, clinical, and cognitive characteristics of T2DM patients with different genotypes.

	GG (101)	GC (89)	CC (43)	*P*
Age (year)	60 (55-67)	60 (53-67)	56 (52-64)	0.210^‡^
Female (%)	36 (40.4)	35 (34.7)	20 (46.5)	0.442^§^
Education (year)	10 (9-12)	11 (9-12)	10 (9-12)	0.914^‡^
BMI (kg/m^2^)	24.93 (22.87-27.09)	14.09 (22.08-26.21)	24.62 (23.60-25.88)	0.318^‡^
DM duration (year)	10 (6-15)	10 (8-16)	10 (6-15)	0.768^‡^
HBP duration (year)	6 (0-16)	3 (0-10)	1 (0-11)	0.230^‡^
Smoking (%)	33 (37.1)	36 (35.6)	13 (30.2)	0.737^§^
Insulin usage (%)	48 (53.9)	60 (59.4)	28 (65.1)	0.442^§^
Metformin usage (%)	56 (62.9)	59 (58.4)	36 (83.7)	0.013^§^^∗^
HbA1c (%)	8.6 (7.6-10.6)	8.7 (7.8-10.5)	8.6 (7.7-10.2)	0.797^‡^
FPG (mmol/l)	7.40 (6.35-10.00)	7.70 (6.61-9.47)	8.00 (6.67-10.20)	0.257^‡^
TG (mmol/l)	1.48 (1.03-2.41)	1.52 (0.90-2.67)	1.67 (1.08-2.25)	0.734^‡^
TC (mmol/l)	4.93 ± 1.06	4.53 ± 1.20	3.94 ± 1.02	<0.001^†^^∗^
HDL (mmol/l)	0.93 (1.09-1.31)	1.10 (0.99-1.28)	1.03 (0.87-1.27)	0.341^‡^
LDL (mmol/l)	2.96 (2.50-3.70)	2.77 (2.22-3.53)	2.14 (1.76-2.71)	<0.001^‡^^∗^
ApoA1	1.12 ± 0.28	1.11 ± 0.28	1.13 ± 0.33	0.869^†^
ApoB	0.82 (0.71-0.97)	0.81 (0.64-1.01)	0.72 (0.59-0.85)	0.005^‡^^∗^
LPa	143 (69-285)	149 (79-318)	111 (70-227)	0.339^‡^
MoCA	26 (22-27)	26 (24-27)	27 (25-28)	0.027^‡^^∗^
DST	12 (11-13)	11 (10-13)	12 (11-13)	0.158^‡^
VFT	15 (13-18)	16 (14-19)	16 (14-20)	0.123^‡^
CDT	4 (3-4)	4 (3-4)	4 (3-4)	0.082^‡^
TMTA	61 (50-101)	58 (48-71)	57 (047-79)	0.109^‡^
TMTB	160 (119-252)	148 (115-189)	128 (80-195)	0.016^‡^^∗^
AVLT-IR	17 (14-21)	18 (15-21)	18 (15-23)	0.391^‡^
AVLT-DR	5 (4-7)	6 (5-7)	6 (05-8)	0.144^‡^
LMT	9.58 ± 4.76	9.24 ± 4.10	10.27 ± 4.68	0.461^†^

BMI: body mass index; DM: diabetes mellitus; HBP: high blood pressure; FPG: fasting plasma glucose; TG: triglycerides; TC: total cholesterol; LDL: low-density lipoprotein cholesterol; HDL: high-density lipoprotein cholesterol; ApoA1: apolipoprotein A1; ApoB: apolipoprotein B; LPa: lipoprotein A; MoCA: Montreal Cognitive Assessment; DST: Digit Span Test; VFT: Verbal Fluency Test; CDT: Clock Drawing Test; TMTA: Trail Making Test-A; TMTB: Trail Making Test-B; AVLT-IR: Auditory Verbal Learning test-immediate recall; AVLT-DR: Auditory Verbal Learning test-delayed recall; LMT: logical memory test; MCI: individuals with mild cognitive impairment; Non-MCI: individuals without mild cognitive impairment. The data are presented as *n* (%), the mean ± SD, or the median (interquartile range) unless otherwise specified. ^†^One-way ANOVA was employed for normally distributed variables. ^‡^The Kruskal-Wallis test was employed for asymmetrically distributed variables. ^§^The chi-squared test was employed for categorical variables. ^∗^*P* < 0.05.

**Table 4:** TC, LDL, ApoB, MoCA, adjusted MoCA, and TMTB in two different genotypes.

	GC vs. GG	CC vs. GC	CC vs. GG
	*P*	*P*	*P*
TC	0.013^†^^∗^	0.004^†^^∗^	<0.001^†∗^
LDL	0.025^‡^^∗^	0.002^‡^^∗^	<0.001^‡^^∗^
ApoB	0.340^‡^	0.010^‡^^∗^	0.001^‡^^∗^
MoCA	0.613^‡^	0.024^‡^^∗^	0.009^‡^^∗^
TMTB	0.108^‡^	0.109^‡^	0.005^‡^^∗^

TC: total cholesterol; LDL: low-density lipoprotein cholesterol; ApoB: apolipoprotein B; MoCA: Montreal Cognitive Assessment; TMTB: Trail Making Test-B. ^†^One-way ANOVA was employed for normally distributed variables. ^‡^The Kruskal-Wallis test was employed for asymmetrically distributed variables. ^∗^*P* < 0.05.

**Table 5:** Relationships between TC, LDL, and ApoB levels and neuropsychological test scores.

	Model^1^		Model^2^		Model^3^	
	*R*	*P*	*R*	*P*	*R*	*P*
MoCA	-0.069	0.350	-0.072	0.286	-0.052	0.437
TMTB	0.145	0.030^∗^	0.193	0.004^∗^	0.021	0.750

^1^Correlation between TC and MoCA (or TMTB) adjustment by age, education level, gender, DM duration, HBP duration, smoking, insulin usage, and metformin usage. ^2^Correlation between LDL and MoCA (or TMTB) adjustment by age, education level, gender, DM duration, HBP duration, smoking, insulin usage, and metformin usage. ^3^Correlation between ApoB and MoCA (or TMTB) adjustment by age, education level, gender, DM duration, HBP duration, smoking, insulin usage, and metformin usage. TC: total cholesterol; LDL: low-density lipoprotein cholesterol; ApoB: apolipoprotein B; MoCA: Montreal Cognitive Assessment; TMTB: Trail Making Test-B. ^∗^*P* < 0.05.

**Table 6:** Multiple linear regression analysis of the factors influencing TMTB.

	Model^1^		Model^2^	
	*β*	*P*	*β*	*P*
Age	1.670	0.006^∗^	1.693	0.005^∗^
Gender	23.573	0.031^∗^	23.756	0.027^∗^
Education	-4.996	0.001^∗^	-5.097	<0.001^∗^
BMI	-1.730	0.215	-1.777	0.198
DM duration	0.454	0.557	0.388	0.630
HBP duration	1.221	0.009	1.263	0.009
Smoking	-0.592	0.957	-0.252	0.981
Insulin usage	-9.854	0.270	-9.314	0.293
Metformin usage	-8.651	0.362	-9.832	0.295
TC or LDL	8.416	0.030^∗^	13.704	0.005^∗^

^1^Independent variables entered included age, education level, gender, DM duration, HBP duration, smoking, insulin usage, metformin usage, and TC. ^2^Independent variables entered included age, education level, gender, DM duration, HBP duration, smoking, insulin usage, metformin usage, and LDL. TC: total cholesterol; LDL: low-density lipoprotein cholesterol; BMI: body mass index; DM: diabetes mellitus; HBP: high blood pressure. ^∗^*P* < 0.05.

## Data Availability

All data used to support the findings of this study are available from the corresponding author upon request.
